# Anti-HIV-1 activity of *Sargassum swartzii* a marine brown alga

**DOI:** 10.1186/1471-2334-14-S3-E43

**Published:** 2014-05-27

**Authors:** Dinesh Subramaniam, Thangam Menon, Hanna Luke Elizabeth, Soumya Swaminathan

**Affiliations:** 1 Department of Microbiology, Dr ALM PG IBMS, University of Madras, Chennai, India; 2 Department of Clinical Research, National Institute for research in Tuberculosis (NIRT), Chennai, India

## Background

Currently available antiretrovirals are relatively expensive and have side effects. Hence, there is need to develop novel drugs against HIV. The present study was planned to investigate the anti-HIV activity of extracts of the marine brown algae *Sargassum swartzii.*

## Methods

The HIV inhibitory activity of aqueous and methanolic extracts of *S. swartzii* was determined on laboratory adapted strains of HIV-1 clades C and A by indirectly measuring reduction in HIV-1 gag p24 antigen levels and inhibition of HIV-1 RT activity in supernatants of virus infected donor peripheral blood mononuclear cell (PBMC) cultures grown in varying concentrations of the extracts. Phytochemical analysis and cytotoxicity of the extracts was also undertaken.

## Results

Results showed that both aqueous and methanolic extracts of *S. swartzii* had an inhibitory effect on HIV-1clade C, and the response was dose dependent. At a concentration of 0.39µg/ml, aqueous and methanolic extracts of *S. swartzii* demonstrated 71.1±2% and 74.7±2.9% inhibition of virus production, respectively. A similar response was observed with a HIV-1 clade A primary isolate. The extracts also showed inhibition of RT activity, and no cytotoxicity on human PBMC. Phytochemical analysis revealed that the aqueous extract of *S. swartzii* contained sulfate and uronic acid, while the methanolic extract contained hexadecanoic acid. These compounds could be responsible for the anti-HIV-1 activity.

## Conclusion

*S. swartzii* extracts significantly inhibited HIV-1 virus suggesting that it may be useful in therapeutics. This study is the first report to show that *S. swartzii* possesses compounds with anti HIV-1 activity.

